# Do Individuals Use Nutrition Labels on Food Packages to Make Healthy Choices? Testing the Dual-Process Model in Two Laboratory-Based Experiments

**DOI:** 10.3390/nu14183732

**Published:** 2022-09-10

**Authors:** Xiaoyan Li, Qi Wang, Chun-Qing Zhang

**Affiliations:** Department of Psychology, Sun Yat-Sen University, Guangzhou 510006, China

**Keywords:** nutrition label, nutritional information, decision-making, dual-process system, time constraints

## Abstract

Nutrition labels on food packages are designed to assist consumers in making healthy decisions. Based on the model of a dual-process system, the current study examined how people might be affected by nutrition labels and consuming contexts when making choices about healthy foods. Using four types of nutrition labels (i.e., the NuVal label, 5-Color nutrition label, traffic light label, and daily value label), participants were instructed to choose the healthier foods with or without time constraints in two experiments. In Experiment 1, participants were presented with pairs of foods accompanied by the same type of nutrition labels to measure the efficiency of their health evaluation. In Experiment 2, two types of labels with inconsistent nutritional information were presented to participants simultaneously to measure their preference regarding the nutrition labels. Findings of the current study support the notion that the traffic light label is advantageous in terms of both the efficiency of, and preferences regarding, nutrition judgment, especially with time constraints. When there was only one type of nutrition label, participants made decisions fastest and most accurately when observing the NuVal label, regardless of time constraints. Overall, the reliable interactions between the time constraints and patterns of nutrition labels have theoretical implications for the appeal-based heuristics and rational-based processing when making health-related food decisions.

## 1. Introduction

Nutrition labels on food packages are designed to standardize and communicate the nutritional information of foods. Healthy organizations and the food industry assume that the visualized nutrition labeling systems are an effective channel for informing customers about nutritional ingredients and affecting their food consumption decisions. However, food choice might be affected by various factors, such as the features of the foods themselves, the designation of food packages, individual differences among the customers, or the purchasing circumstances [1,2,3]. Although there are existing studies examining choices regarding healthy food [1,4,5,6], it is unclear how the aforementioned factors interact with each other when making food choices. From the perspective of cognitive processing, the present study therefore aimed to examine the effects of nutrition labels and consuming contexts on the judgment of the healthiness of foods. 

The widely used nutritional labeling systems are normally categorized using diverse criteria [7,8,9,10]. For example, nutrition labels using non-directive, semi-directive, and directive labeling systems are based on the extent of the overall healthiness of the food [11]. In line with the classification system proposed by Hersey et al. [7], nutrition labels are categorized as specific systems and summary systems. Specific labeling systems present the amount of each nutrient in the food, such as sugar, protein, or energy, while the summary labeling systems present a general nutritional score calculated using certain regulations based on all the contained nutrients. There are even summary labels using a simpler pattern, such as those showing a “tick” or an “eco-label” sign on the food packages [5,6,7].

Each nutrition labeling system has its merits. By presenting various nutrition labels, researchers have compared the accuracy, response times, and comprehension of labels, and evaluated the degree of healthiness of certain foods [8,11,12,13,14,15,16,17,18]. However, the findings of previous studies are inconsistent and inconclusive. On the one hand, consumers prefer simple summary labels and tend to rely on them to form judgments, rather than relying on a specific labeling system [8,15,16,19,20,21,22]. For example, Ducrot et al. [8] required participants to choose foods for one week in a virtual store and found that individuals preferred food products with a single-colored nutrition label inferring a high nutritional quality. Researchers also investigated the attitudes towards and comprehension of five types of nutrition labels in twelve countries and found the Nutri-Score label, which belongs to the summary labeling system, was preferred [22]. A summary labeling system can effectively reduce the complexity of decision-making in the process of food selection [12]. On the other hand, however, consumers preferred the specific system of nutrition labels when they were particularly concerned about their intake of certain nutrients [13,18]. Recent studies also support the efficiency of, and preference for, the traffic light label as a kind of specific system label when capturing attention and making food decisions, though it may not facilitate the understanding of nutritional information [23,24,25]. Comparing the summary and specific labeling systems, the review of Hersey et al. [7] identified six out of ten studies showing that the specific labeling system is more useful for identifying nutrients and evaluating food healthiness. In comparison, three other studies indicated that consumers preferred the summary labeling system, while one study did not show any differences between the two systems. 

To sum up, it seems that the inconclusive findings regarding the utility of nutrition labels might be due to diverse paradigms or tasks adopted in previous studies. The dual-process model, which includes the heuristic system and analytical system, could be used to explain the inconsistent findings from the perspective of cognitive effort [11,26,27]. The heuristic system is automatic and requires limited working memory or attention resources, while the analytical system is affected by conscious control and effortful processing [28,29]. Accordingly, the current study focused on the classification of specific and summary labeling systems, as they may require different cognitive loads during the processing of nutritional information. This approach is in line with the two-route food choice model of nutrition label processing proposed by Ma and Zhuang [11]. This model included a rational route for accessing healthier foods and a heuristic route that is affected by individual preferences and purchasing contexts. Consequently, for the task of evaluating food healthiness, participants appeared to form their judgments by computing the nutritional information present on the labels. The more the pattern of a nutrition label matches the final decision of health evaluation, the higher the probability that the participants would use that very type of label was. It should be noted that, when the evaluation is based on two or more types of nutrition labels, responses may reflect the least cognitive effort required for the analytical system or the heuristic decision-making based on the preference of, or trust in, either of label types [30,31,32]. Furthermore, consuming contexts, involving completion time, may also interact with the processing of nutrition labels, taking the cognitive effort for the heuristic and analytical systems into consideration. Under time pressure, limited cognitive resources may lead to the high efficiency of, and preference for, a heuristic label or simple summary label requiring the least cognitive effort. 

Therefore, in the current study, we examined the efficiency of, and preference for, nutrition labels in different consuming contexts by adopting different paradigms of nutrition evaluation tasks. Although the daily value label, as known as the nutrition facts panel (NFP), is the only pattern of nutrition label used in the current Chinese food industry, more than 70% of consumers never use nutrition labels to evaluate food healthiness during purchases [33]. According to previous surveys on the comprehension of nutrition labels by Chinese consumers, the majority of consumers have difficulty understanding and using the daily value label to evaluate food healthiness [33,34,35]. That said, it could be assumed that Chinese customers would have difficulty fully understanding daily value labels, as well as other types of nutrition labels. This means that participants in the current study had roughly equivalent levels of familiarity with the various types of nutrition labels. 

To examine the cognitive processing involved in the summary and specific labeling systems, we conducted two experiments by employing a nutrition evaluation task. In Experiment 1, participants were required to compare the healthiness of paired foods using the same type of nutrition label under regular or time-constraint conditions. Given that the summary labels straightforward nutrition level information, it was expected that participants might be more likely to make their decisions using the summary labels under time pressure (H1), while the advantages of summary labels may not be significant without the time constraints compared to the specific labeling system (H2). In Experiment 2, participants evaluated food healthiness using two types of nutrition labels, which provided conflicting nutritional information. We assumed that participants would make decisions based on their preferences differently in time-constraint as opposed to regular conditions. Theoretically, we expected that Experiment 1 would demonstrate the cognitive efforts involved when processing nutrition labels, while the responses given in Experiment 2 would reflect the heuristic preference for nutrition labels.

## 2. Experiment 1

### 2.1. Method

#### 2.1.1. Participants

A prior power analysis was performed using G*Power 3.1.9.7 [36], indicating that a minimum of 23 participants were required with an effect size f of 0.25, α error probability of 0.05, and power (1-β error probability) of 0.95 in a repeated measure analysis of variance (ANOVA) using the within-subject experiment design. Considering potential dropouts or problems due to experimental errors during the tests, 44 college-age volunteers (23 males, 21 females, *M*_age_ = 19.64 years old, *SD* = 1.43) were recruited as participants. Due to an accidental termination of the experimental program, the data for two participants (one male and one female) could not be used for the analysis. Each participant signed an informed consent declaration form before the experiment, and monetary compensation was provided after the experiment. The present study was approved by the Institutional Review Board of the Department of Psychology, Sun Yat-Sen University, and was performed following the relevant national and international ethical guidelines and regulations. 

#### 2.1.2. Materials

**Figure 1:**
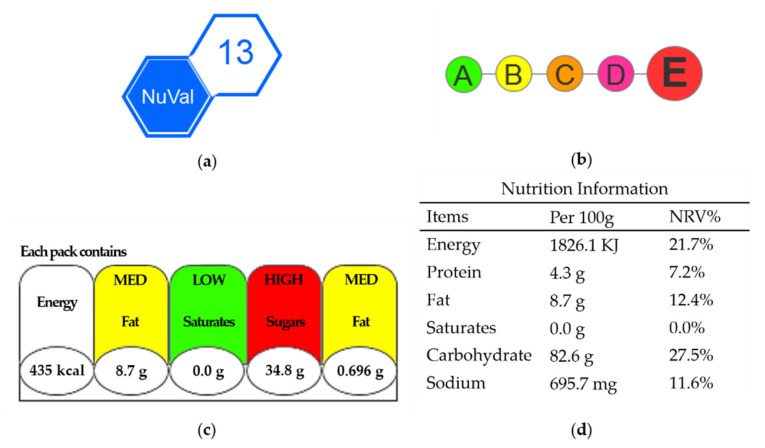
Nutrition labels. (**a**) The NuVal label; (**b**) the 5-color nutrition label (5-CNL); (**c**) the traffic light (TL) label; (**d**) the daily value (DV) label.

We used four types of nutrition labels to represent the health levels of food products (see Figure 1). The NuVal and 5-color nutrition label (5-CNL) were assigned to the summary system, while the traffic light (TL) and daily value (DV) were assigned the specific system. The NuVal label represents the nutrition level using a single score in the range of 1 to 100 (NuVal Attribute Program, 2017). The larger the NuVal score is, the healthier the specific food is deemed to be by the NuVal system. The 5-CNL label defines food nutrition using five levels (A/B/C/D/E) [37]. Level A is visualized in green and represents the healthiest level, while Level E is the lowest health level, shown in red. The TL label presents the amount of each nutrient in grams per unit of the food, including fat, saturated fat, sugar, and sodium, as well as the corresponding calories in kilo joules (KJ) [37,38]. There are also three levels denoting the amount of nutrients on the TL label, including low, medium, and high in the colors of green, amber, and red, respectively. Similarly, the DV label presents the amounts of nutrients in grams and with the nutrient reference value (NRV) per 100 g of the specific food, including protein, fat, saturated fat, carbohydrate, and sodium, and the corresponding calories in KJ. However, the DV label demonstrates the nutrient information only through a monochrome table of numerical values [39].

As the NuVal score is a summarized value defined by the comprehensive assessments of various foods, it is straightforward to compare food healthiness levels on the basis of its explicit granularity. Thus, ten types of food were selected based on their NuVal scores, which were evenly distributed in the range of 1 to 100. The ten labels of the selected foods comprised 45 label pairs, of which 17 pairs were kept aside for the nutrition evaluation task using the differences in their NuVal scores in the range of 20 to 70. Then, we matched the 5-CNL, TL, and DV labels to the corresponding NuVal scores and created visual images for each type of labeling system. The 17 label pairs for each type of nutrition label were assigned to the corresponding pairs of food images. There were 68 label pairs in total that were used in the formal study. We used artificial images of food packages to eliminate the effects of branding and familiarity. The pairs of food images presented in one experiment were designed to share the same category (e.g., cookies), context, and fake brand. The differences between the pairs of foods were based on the foods’ appearances or names.

#### 2.1.3. Design

The experiment used a 2 (time constraint: with and without) × 2 (labeling systems: summary and specific) within-participant design. The time constraint variable was manipulated by asking participants to respond within 1600 ms in the time-constraint conditions, while there were no time limits for the non-time-constraint conditions. Participants were presented with 68 pairs of food images, as well as their nutrition labels, both with and without time constraints. Thus, participants completed a total of 136 trials in the experiment with an additional 20 trials for practice. The positions of food images were distributed equally among the participants. The accuracy and response time results of each trial were collected by the program. We also compared the reliability of responses between the with- and without-time-constraint conditions. When participants made the same response for a specific food pair under the with- and without-time-constraint conditions, it would be coded as 1. Otherwise, the program would code the result as zero.

#### 2.1.4. Procedure

**Figure 2:**
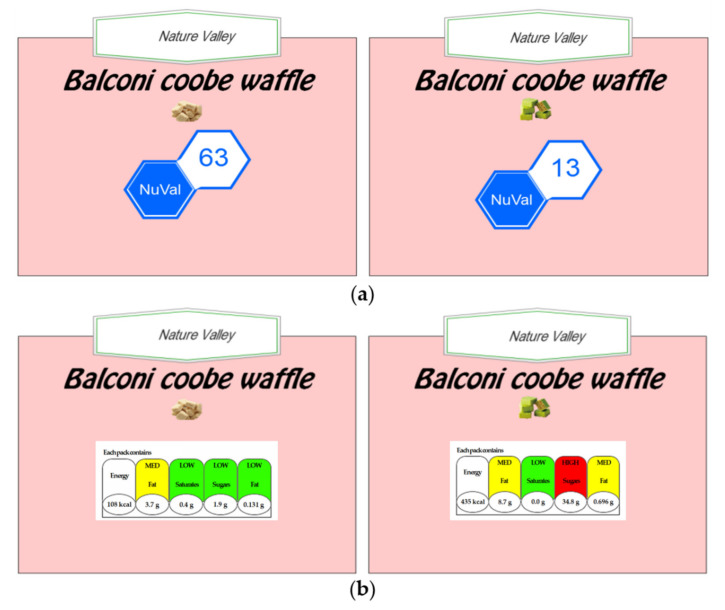
Examples of food images presented in Experiment 1: (**a**) illustrates a pair of waffles accompanied by their respective NuVal labels; (**b**) illustrates the paired foods with the traffic light labels.

Each participant completed the practice and formal session individually. For each trial, participants would be presented with a pair of food images, including the corresponding names and nutrition labels of the same labeling system (Figure 2). Participants were asked to choose which one of the paired foods was more nutritious and healthier. Feedback on the responses was provided to participants in the practice session to validate and correct their understanding of the nutrition labels. There was no feedback in the formal experiment.

The formal experiment was divided into two blocks. To avoid the confounding effect of the practice, the block with time constraints was always run first. In the first block, participants were required to respond within 1600 ms. This time constraint was set based on a pilot study, which suggested that participants would not feel the time pressure if it lasted more than 2000 ms or that they might not have enough time to form responses if it lasted less than 1500 ms. A timer visually counted down the time during the trials in this block. The second block, which did not involve time constraints, required participants to consider their choices carefully before they made their responses.

After the two experimental blocks, each participant completed the Chinese version of the General Nutrition Knowledge Questionnaire (GNKQ) [40] to measure their knowledge about nutrition and healthy eating.

### 2.2. Results

Based on the responses of the GNKQ, participants showed a chance level of accurate nutritional knowledge. Thus, the level of nutritional knowledge would not be included in the further analyses. We examined the effects of the labeling system and consuming context. The accuracy rate and response time of each participant were submitted using a 2 (time constraint: with and without) × 2 (labeling systems: summary and specific) repeated measure ANOVA. The statistical analysis was performed using SPSS 22.0. For the accuracy, the main effect of the time constraint was significant, *F*(1, 41) = 34.41, *p* < 0.001, *η*^2^ = 0.456. Participants performed more accurately in the trials without time constraints (*M* = 0.973, *SD* = 0.039) compared to those with time constraints (*M* = 0.922, *SD* = 0.083). The main effect of the labeling system was not significant in terms of the accuracy, as well as the interaction (*ps* > 0.50). For the response time, the main effect of the time constraint was also significant, *F*(1, 41) = 39.73, *p* < 0.001, *η*^2^ = 0.50. Participants not under time constraints responded more slowly (*M* = 1581.83, *SD* = 661.50) than those under time constraints (*M* = 1030.05, *SD* = 665.47). The main effect of the labeling system was significant, *F*(1, 41) = 143.62, *p* < 0.001, *η*^2^ = 0.78. Participants responded faster using the summary system labels (*M* = 1145.17, *SD* = 499.05) compared to the specific system labels (*M* = 1466.71, *SD* = 563.93). The two-way interaction between the time constraints and labeling systems was also significant, *F*(1, 41) = 49.41, *p* < 0.001, *η*^2^ = 0.55. The simple effect analysis showed significant differences between the two labeling systems with and without time constraint (*p*s < 0.05), while the difference in the latter condition was shown to a greater extent. A one-way repeated ANOVA was used to explore the reliability of the decisions under the time-constraint and non-time-constraint conditions, and it revealed the main effect of the labeling system, *F*(1, 41) = 8.71, *p* = 0.005, *η*^2^ = 0.18. The reliability of the summary system labels (*M* = 0.90, *SD* = 0.130) was higher than that of specific system labels (*M* = 0.85, *SD* = 0.127). Overall, the summary labeling system demonstrated a higher accuracy, shorter response time, and higher reliability compared to the specific labelling system. 

**Figure 3:**
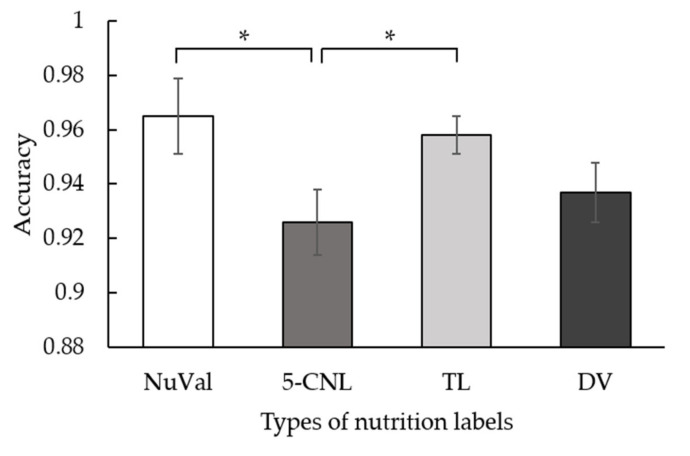
The main effect of the type of nutrition label on accuracy in Experiment 1. The error bar stands for the standard error. * is *p* < 0.05. NuVal indicates the NuVal label. 5-CNL indicates the 5-color nutrition label.TL indicates the traffic light label. DV indicates the daily value label.

To analyze the effect of the type of the nutrition label, we conducted a 2 (time constraint: with and without) × 4 (types of nutrition labels: NuVal, 5-CNL, TL, and DV) repeated measure ANOVA. Due to the assumption of Mauchly’s sphericity, we adjusted the degrees of freedom via the Greenhouse–Geisser procedure. For accuracy, the main effect of the time constraint was significant, *F*(1, 41) = 34.83, *p* < 0.001, *η*^2^ = 0.459. Participants performed more accurately in the trials without time constraints (*M* = 0.973, *SD* = 0.039) compared to those with time constraints (*M* = 0.922, *SD* = 0.083). The main effect of the nutrition labels was significant, *F*(2.45, 100.62) = 3.10, *p* = 0.040, *η*^2^ = 0.070 (see Figure 3). The accuracy rate obtained using the 5-CNL label was lower than that obtained using the NuVal label (*MD* = −0.04, *p* = 0.029, *95% CI* = [−0.074, −0.004]) and the TL label (*MD* = −0.03, *p* = 0.023, *95% CI* = [−0.060, −0.005]). Yet, the other comparisons were not significant. The with-time-constraint condition led to a remarkably low accuracy rate when using the 5-CNL and DV labels, though the two-way interaction between the time constraint and types of nutrition labels was not significant, *F*(2.23, 91.24) = 2.88, *p* = 0.056, *η*² = 0.066 (see Table 1 for means).

**Figure 4:**
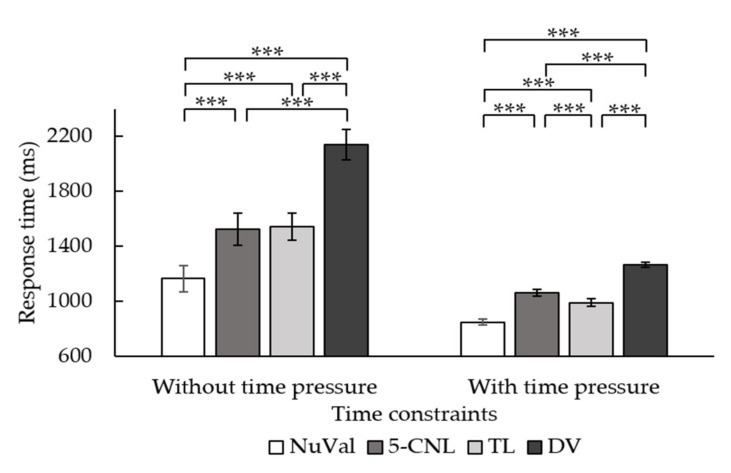
Interaction between the time constraints and types of nutrition labels in affecting the response time in Experiment 1. The error bar stands for the standard error. *** is *p* < 0.001.

Regarding the response time, the main effect of the time constraint was significant, *F*(1, 41) = 37.65, *p* < 0.001, *η*^2^ = 0.479. Participants who were not under time constraints responded more slowly (*M* = 1581.83, *SD* = 661.50) than those under time constraints (*M* = 1030.05, *SD* = 665.47). The main effect of the type of nutrition label was significant, *F*(3, 123) = 99.44, *p* < 0.001, *η*^2^ = 0.708. The two-way interaction between the time constraints and types of nutrition labels was significant, *F*(2.46, 100.65) = 21.89, *p* < 0.001, *η*^2^ = 0.348 (see Table 1 for means). The simple effect analysis revealed that, when the time was not constrained, participants responded the fastest using the NuVal label and the slowest using the DV label. The differences between the 5-CNL label and the TL label were not significant. However, it was interesting that, under time constraints, participants responded more slowly using the 5-CNL label than the TL label, as Figure 4 illustrates.

A one-way repeated ANOVA of the reliability of the four types of nutrition labels showed a significant main effect, *F*(2.36, 96.55) = 20.73, *p* < 0.001, *η*^2^ = 0.336. A post hoc analysis showed that the reliability of the NuVal label was the highest and significantly differed from the 5-CNL label (*MD* = 1.33, *p* < 0.01, *95% CI* = [0.77, 1.90]), the TL label (*MD* = 1.357, *p* = 0.002, *95% CI* = [0.54, 2.17]), and the DV label (*MD* = 3.31, *p* < 0.001, *95% CI* = [2.31, 4.31]). Additionally, the reliability of the DV label was lower than that of the 5-CNL label (*MD* = −1.98, *p* < 0.001, *95% CI* = [−2.98, −0.97]) and that of the TL label (*MD* = −1.95, *p* < 0.001, *95% CI* = [−2.82, −1.08], while the latter two did not show significant differences (*MD* = 0.024, *p* = 0.952). Consistent with the results of the accuracy and response time, the NuVal and DV label showed the highest and the lowest reliability, respectively.

### 2.3. Discussion

As expected, when participants were asked to evaluate food healthiness using nutrition labels, trials using the NuVal label showed the highest accuracy and the shortest response time. This result supported our H1, that participants would attempt to deploy the least cognitive effort in order to evaluate the nutrition level, leading to the advantage of the summary labeling system, especially in the case of the NuVal labels. Considering that the NuVal value is positively correlated with food healthiness, participants may spontaneously and steadily use the NuVal label to perform the health evaluations, especially in a situation involving time constraints. As the college-age participants were quite familiar with the numerical system, they formed judgments using the NuVal labels rapidly and automatically. In addition, as participants received feedback on their responses in the practice block, they may have realized that the criteria of the NuVal label are generally simple and stable in the formal blocks. If participants used the NuVal label to evaluate food healthiness, it appears that they logically acquired the best performance and the highest reliability in both the time-constraint and non-time-constraint conditions. 

On the other hand, trials using the DV label showed the lowest accuracy and the longest response time. Our H2 is not supported, as the DV label showed the worst performance even without time constraints. While the NuVal label offers the simplest structure of the nutritional information among the labels, it might be difficult for participants to understand the DV label due to its complex pattern of numerical values and unclear format. Participants with little prior nutritional knowledge require more cognitive effort to interpret the nutrient information on DV labels, especially under time constraints. The interaction between the time constraint factor and labeling systems also provided evidence for this process by showing that a much longer time was required to understand the specific labeling system when participants were freely making their responses.

Furthermore, the different performances of the nutrition labels suggested that the processing of the labeling system was affected by both the pattern of the label and the consuming context. For example, the TL label, as a type of specific labeling system, demonstrated a surprisingly strong performance even under time constraints. Inconsistent with our hypothesis, however, the use of the 5-CNL label, as a summary labeling system, led to an even longer response time than the TL label under time constraints. Although the 5-CNL label illustrates the nutritional levels using a summarized pattern, with five typical colors and letters, participants might be confused about the meaning of the symbols. They may keep changing their criteria and use a strategy of trial-and-error, leading to the low accuracy and long response times observed when using the 5-CNL label. However, for the TL labels, participants were able to view the rating of each specific nutrient directly on the visualized label, indicated by its corresponding color. The text and color provide redundant information, leading to a decrease in the cognitive workload required and advantages for the nutrition evaluation [41,42,43,44]. To further investigate the possible advantage of the design of TL labels, Experiment 2 explored differences between the four types of nutrition labels from the perspective of preference when judging the food healthiness with or without time constraints.

## 3. Experiment 2

The results of Experiment 1 suggest that the summary labeling system may have advantages in reducing cognitive loads when evaluating healthiness compared with the specific labeling system. Nevertheless, there was an exception, namely, that participants performed better using the TL label than the 5-CNL label. In Experiment 2, we designated a different paradigm to compare the efficiency and preferences between the four types of nutrition labels. Similarly, we hypothesized that participants would be affected by the time constraints and be inclined to use the summary labeling system rather than the specific labeling system under time constraints (H3). Furthermore, according to previous research, we expected the clear efficiency of, and preference for, the TL label, as it can capture attention more readily than the other types of labels [24,25,45,46,47,48]. Thus, if the participants preferred the TL labels, as in previous studies, we could expect that they would continue to judge food healthiness based on the TL labels rather than other types of labels in Experiment 2 (H4).

### 3.1. Method

#### 3.1.1. Participants

The prior power analysis using G*Power 3.1.9.7 suggested that a minimum of 23 participants were required for the effect size f of 0.25, with an α error probability of 0.05 and power (1-β error probability) of 0.95 [36]. To account for potential dropouts and experimental errors during the test, 51 college-age volunteers (19 males, 32 females, *M_age_* = 20.34 years old, *SD* = 1.64) took part in the experiment individually. Each participant signed an informed consent declaration form before the experiment and received monetary compensation after the experiment. The data of one female participant could not be used due to the accidental termination of the experimental program.

#### 3.1.2. Materials and Design

**Figure 5:**
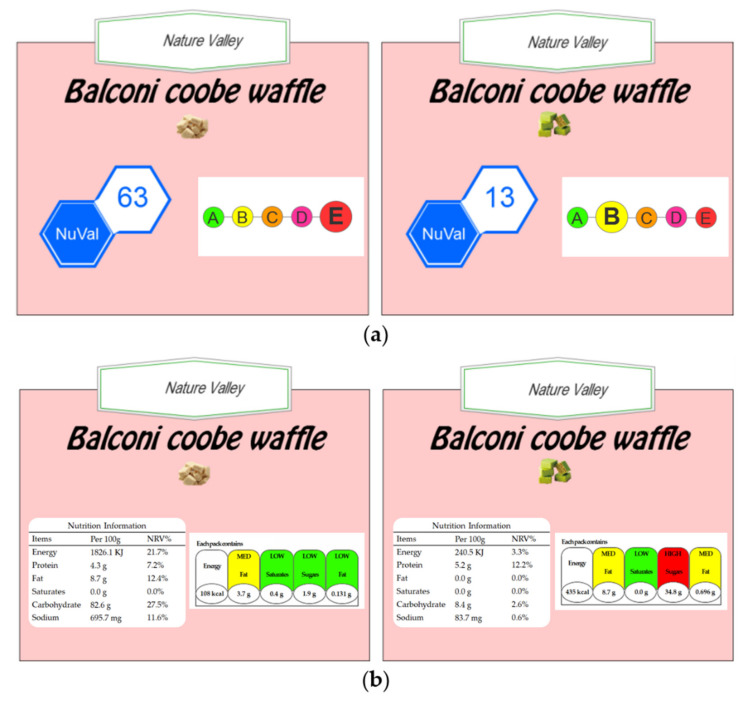
Examples of food images presented in Experiment 2: (**a**) illustrates a pair of waffles accompanied by both NuVal labels and 5-CNL labels; (**b**) illustrates the paired foods with both traffic light labels and DV labels.

Eleven food pairs were randomly selected from the 17 food pairs used in Experiment 1. The four types of nutrition labels were classified into six types of combinations: NuVal-5-CNL, NuVal-TL, NuVal-DV, 5-CNL-TL, 5-CNL-DV, and TL-DV. Each food pair was presented with two types of nutrition labels, with participants choosing from the six combinations above (Figure 5). Usually, the evaluation of a specific food’s nutritional level is consistent across different nutrition labeling systems. To examine the preference for nutrition labels, we manipulated the label combinations in the same trial so that they showed inconsistent nutritional information. When a pair of foods, such as M and N, was presented, an immediate NuVal label and a 5-CNL label were presented at the same time. The nutritional levels on the NuVal label demonstrated an opposite tendency to that on the 5-CNL label. That is, while the NuVal labels inferred a higher nutritional level of M than that of N, the 5-CNL labels inferred the opposite. We considered that, if the participant considered M as the healthier food, then they would make their decisions based on the NuVal labels. Otherwise, they would choose N as the healthier food based on the 5-CNL labels.

#### 3.1.3. Design and Procedure

The experiment used a 2 (time constraint: with and without) × 4 (types of nutrition labels: NuVal, 5-CNL, TL, and DV) mixed design. The time constraint variables were in line with the design of Experiment 1. Participants were presented with 66 food pairs in blocks with and without time constraints, using 11 pairs for each label combination, respectively. In total, participants completed 132 trials in the experiment, in addition to 20 trials for practice. The positions of the food images and labels were distributed equally among the participants. The response time to, and trust in, the labels of each trial were collected by the program. After participants chose the healthier food in each trial, the program recorded which label was used to make the decision. The number of times that choosing a type of nutrition label were coded as the indicator of preference for the very type of label.

The procedures of Experiment 2 are the same as those of Experiment 1.

### 3.2. Results

**Figure 6:**
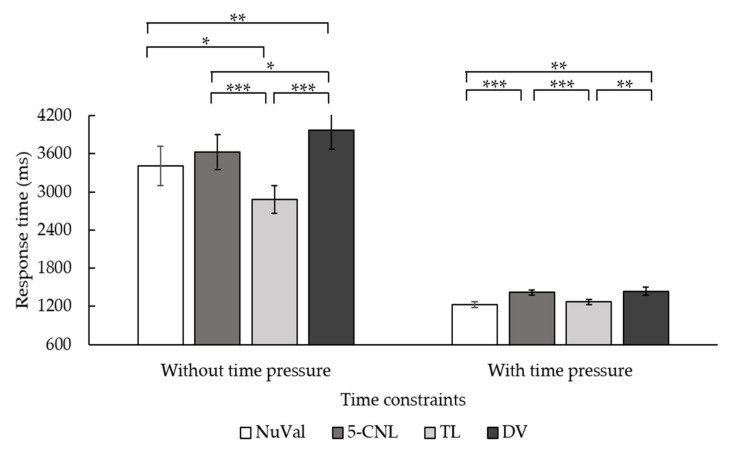
Interaction between the time constraints and types of nutrition labels in terms of the response time in Experiment 2. The error bar stands for the standard error. * represents that *p* < 0.05. ** represents that *p* < 0.01. *** represents that *p* < 0.001.

We removed the data of the participant as the data file was damaged due to technological reasons. Similar to the findings of Experiment 1, the accuracy of the nutritional knowledge survey for participants did not higher than the chance level, so this was not included in the further analyses. The response times were subject to a 2 (time constraint: with and without) × 4 (types of nutrition labels: NuVal, 5-CNL, TL, and DV) repeated measure ANOVA. The results showed that the main effect of the time constraints was significant, *F*(1, 37) = 84.15, *p* < 0.001, *η*^2^ = 0.70. Participants who were not under time constraints (*M* = 3471.74, *SD* = 1760.56) responded more slowly than those under time constraints (*M* = 1339.62, *SD* = 302.21). The main effect of the type of nutrition label was significant, *F*(3, 111) = 13.06, *p* < 0.001, *η*^2^ = 0.26. Post hoc tests revealed that participants responded fastest using the TL label compared to other labels (*ps* < 0.05). Participants responded more slowly using the DV label than the NuVal label (*p* = 0.001) but not the 5-CNL label (*p* > 0.05). The two-way interaction between the time constraints and labeling systems was also significant, *F*(3, 111) = 6.63, *p* < 0.001, *η*^2^ = 0.15 (see Table 2 for means). The simple effect analysis revealed that, without time constraints, participants responded the fastest using the TL label than the other three types of labels (see Figure 6). Moreover, they responded slowest using the DV label. However, under time constraints, participants showed different response patterns. They responded faster when using the NuVal and TL label than the 5-CNL label and the DV label. There were no differences between the NuVal and TL, or between the 5-CNL and DV labels. Overall, the TL label led to the fastest responses regardless of the time constraints, while NuVal label led to faster response under time pressure than the 5-CNL and DV labels.

**Figure 7:**
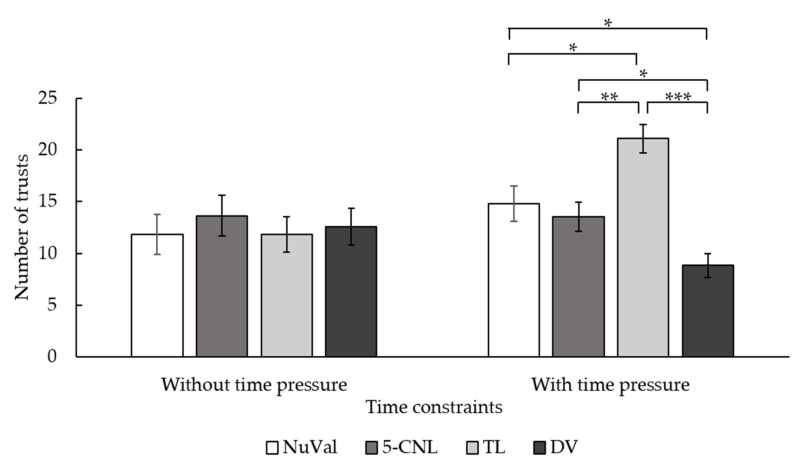
Interaction between the time constraints and types of nutrition labels in terms of the number of times for choosing nutrition label types in Experiment 2. The error bar stands for the standard error. * represents that *p* < 0.05. ** represents that *p* < 0.01. *** represents that *p* < 0.001.

In regard to the preferences for the types of nutrition labels, the results showed that there was no significant difference between the conditions with and without time constraints, *F*(1, 24) = 1.82, *p* = 0.19, *η*^2^ = 0.07. The main effect of the type of nutrition label was significant, *F*(3, 72) = 5.68, *p* = 0.002, *η*^2^ = 0.19. Post hoc tests revealed that, compared to the DV label, participants were more inclined to trust the 5-CNL (*p* = 0.019) and the TL label (*p* < 0.001). The two-way interaction between the time constraints and labeling systems was significant, *F*(3, 72) = 8.12, *p* < 0.001, *η*^2^ = 0.25 (see Table 2 for means). Without time constraints, participants showed no significant preference for any of the diverse nutrition labels. However, when there were time constraints, they relied mostly on the TL label compared to the NuVal, the 5-CNL, and the DV label. Additionally, they preferred the NuVal and the 5-CNL label rather than the DV label. That is, as Figure 7 shows, the TL label and DV label had the highest and lowest preference under time constraints, respectively.

### 3.3. Discussion

The findings of Experiment 2 demonstrated that participants, under time constraints, showed the highest level of trust in the TL label but the lowest level in the DV label. There was no preference for any nutrition label type in the non-time-constraint situation. Without time pressure, participants could make decisions using both types of nutrition labels presented with the food pairs, which may have determined their preference for the labels. Another possible reason for this finding is that participants responded based on their preferences for the specific food product rather than the nutrition labels, especially when participants had little prior knowledge of the diverse nutrition labels [49]. 

The results of response time partially support H3 by showing that the shortest response time was achieved when using the NuVal label under time constraints, as seen in Experiment 1 [50]. However, the TL label also demonstrated the most efficient responses under time pressure, supporting H4. Furthermore, participants who were not under time constraints responded the fastest using the TL label and the slowest using the DV label. While the DV label was the most monotonous type of nutrition label, with the most information, participants would naturally be captured by and respond faster to the colorful nutrition labels than to the monochrome labels [51]. That said, Experiment 2 supported H4, that there is an advantage of the TL labels from the perspectives of both preference and the response time.

## 4. General Discussion

The current study aimed to examine the cognitive processing of nutrition labels from the perspectives of comprehension and preference. Overall, our findings on four nutrition labels, in terms of the decisions and response times, varied across experimental conditions, indicating the reliable interaction between the consuming context and the pattern of the nutrition labels. For the nutrition evaluation task in Experiment 1, participants made their judgments on food healthiness based on one single type of nutrition label. Generally, participants demonstrated the highest efficiency and reliability when using the NuVal labels, belonging to the summary system, while they showed the worst performance and reliability when using the DV labels, belonging to the specific system, in both time-constraint and non-time-constraint conditions. One interesting exception in terms of the time-constraint condition was that participants took less time to process the TL labels than the 5-CNL labels, though the latter illustrated the nutrition level using a summary pattern. In Experiment 2, the preference for, and efficiency of, the nutrition labels was further examined by presenting two types of labels simultaneously with conflicting nutritional information. Although, without time pressure, the participants did not show a marked preference for any of the nutrition labels, they made the most efficient decisions when using the TL labels and the slowest responses with the DV Labels. Consistently, under time constraints, participants trusted the TL label the most and made decisions rapidly, while the DV labels were always the last and the slowest option.

The TL label was the most efficient for evaluating food healthiness, though it did not always lead to the correct response [24,25,45,46,47]. Consistent evidence was found across both experiments, even under time pressure. This is in line with previous studies, indicating that the TL label showed a high efficiency and capacity to capture attention when consumers were identifying healthier foods compared with the monochrome DV label and the colored DV label [23,25,52]. In a previous study, participants scanned bar codes on the products to view their nutritional information, and the study showed the advantage of the TL labels in reducing sugar consumption [8]. It seems that these findings are in contrary to the effect of the cognitive load on information processing, as the specific labeling systems provide detailed information about each nutrient. Meanwhile, consumers generally require finite cognitive effort for purchasing food. For example, consumers, in real life, usually make purchasing decisions within seconds, wherein only limited information on the food packages can be detected and processed [53,54]. We speculated that the reason for the high efficiency of, and preference for, the TL label may lie in the visualized pattern and information demands. Firstly, the TL label combines colors, ranks, and values to illustrate the amounts of particular ingredients, which could assist consumers in encoding the nutritional information in a redundant way [25,46,55]. As mentioned earlier, the visualization by typical colors on TL labels may drive automatically heuristic processing, leading to their high efficiency in the present study. Furthermore, the detailed nutrient information provided by TL labels may also make participants more confident about their responses compared to the summary labels, which provide no detailed evidence to support their choices [47]. Research has also suggested that consumers with a high-level nutrition knowledge are more likely to use inspirational and complex nutrition labels [14]. Thus, the pattern of TL labels, showing nutritional information through both visualized elements and specific relevant texts, could facilitate participants in making their decision with little cognitive effort and a high degree of confidence. Even though participants may not have enough prior knowledge of nutritional information, they still show a strong preference for the TL labels.

The current study supports the advantage of the NuVal label, with a single value, in the nutrition evaluation task [8,12,15,16]. As a summary labeling system, the pattern of the NuVal label is more straightforward in conveying the nutrition level than other kinds of labels. The NuVal label is a 1–100-based type of nutrition label, using only numeric scores to measure the nutrients, which can assist consumers in making decisions in urgent circumstances [50]. Though it may involve the cognitive computation processing, individuals can readily compare the value of the label through numerical processing compared to the specific labeling system [12,15,56]. In contrast, though the DV label is more informative than the NuVal label, it requires more prior nutritional knowledge in order to interpret its complicated pattern [56,57]. When the purchase time is constrained, consumers may not have sufficient cognitive resources to evaluate food healthiness through the rational computation of DV labels [27]. On the other hand, heuristic processing is difficult with the monochrome DV label, as the visualized format may not be prominent enough to facilitate the automatic processing. These valid reasons seem to explain the fact that the DV label was the least favorite nutrition label [11,55].

Surprisingly, the 5-CNL label, as a type of summary system and directional label, did not effectively facilitate the nutritional evaluation, though previous studies suggested the positive effects of the 5-CNL label in directing customers’ attention [8,17,45,58]. Consistent findings were revealed in both Experiments 1 and 2, especially under the time constraint conditions. Unlike the NuVal label, the 5-CNL label illustrates the nutrition level through three dimensions, including the color, size, and symbols. However, the 5-CNL label failed to offer a clear elaboration in order to explain the exact meaning of each dimension, and how the features were combined to identify the nutrition level. Hence, participants had to figure out which symbol on the 5-CNL label indicated the nutrition level when they were first confronted with all five grades in diverse colors. Although the size of the symbol on the 5-CNL label may help consumers to infer the nutrition grade, the prominent colors of the symbols might also stand out and interfere with the processing of the nutrition levels [59]. Consequently, the 5-CNL label, as a summary labeling system, did not show any advantages during the nutrition evaluation in terms of the reliability, preference, or efficiency. The all-in-one display of diverse nutrients on 5-CNL labels does not appear to facilitate either cognitive computation, due to its vague regulations, or heuristic processing, because of the roughly equal prominence of its simple visual features [27].

## 5. Conclusions and Limitations

Overall, the findings of the current study demonstrated the high efficiency and accuracy of the NuVal label in the nutrition evaluation task, where consumed were required to choose the healthier foods under both time-constraint and non-time-constraint conditions. The participants showed a consistent preference for, and high efficiency when using, the traffic light label when two types of nutrition labels were presented simultaneously, especially under time constraints. The interaction between the usage of the nutrition labels and the consuming context provides empirical support for the dual-process system. The preference for the traffic light label may reflect the heuristic processing of visualized patterns as a result of the consumers’ attention being captured by the prominent colors, while the NuVal label may require the least cognitive resource when making rational decisions about choosing healthier foods. Contrary to our expectations, the findings of the current study did not support the effectiveness of the 5-CNL label, as previous research suggested [8,17,45,58]. Future research could further explore the inconsistency in the attitudes toward the 5-CNL label. Further empirical evidence is also needed, which may be acquired by using new approaches, such as the examination of eye-movement trajectories or neural activities in order to disclose the effects of the cognitive load and heuristic preference on the processing of various nutrition labels. 

In addition, the current study only focused on the comprehension processes of nutrition labels, which may not directly determine food choices in real purchases. Importantly, researchers require more empirical evidence about whether or how the understanding of, and preference for, nutrition labels can predict health-related decisions [24,25,45,46,47]. Moreover, the effects of nutrition labels are inevitably affected by the individual differences between customers [1,24,60]. Specifically, because most college students do not need to consume food for the whole family or ensure healthy eating, this may have led to a plausible sample bias regarding the omission of the nutrition labels. Future research could validate the current findings by recruiting a more diversified sample of consumers.

## Figures and Tables

**Table 1:** Mean accuracy and response time results of nutrition labels in Experiment 1.

Measurements	Label	Without Time Constraints			With Time Constraints		
		Mean	SD	95% CI	Mean	SD	95% CI
Accuracy	NuVal	0.98	0.01	[0.96, 1.00]	0.95	0.02	[0.92, 0.99]
	5-CNL	0.96	0.01	[0.94, 0.98]	0.89	0.02	[0.85, 0.93]
	TL	0.98	0.01	[0.97, 0.99]	0.94	0.01	[0.92, 0.96]
	DV	0.98	0.01	[0.96, 0.99]	0.90	0.02	[0.86, 0.94]
Response time	NuVal	1166.06	94.70	[974.81, 1357.30]	851.81	19.52	[812.39, 891.23]
	5-CNL	1525.20	116.99	[1288.94, 1761.46]	1064.17	24.33	[1015.03, 1113.31]
	TL	1544.55	100.41	[1341.76, 1747.34]	992.66	27.10	[937.93, 1047.39]
	DV	2141.54	110.02	[1919.34, 2363.73]	1266.04	19.68	[1226.31, 1305.78]

Note: SD means the standard deviation; NuVal indicates the NuVal label; 5-CNL indicates the 5-color nutrition label; TL indicates the traffic light label; DV indicates the daily value label.

**Table 2:** Mean response time and the number of times choosing a type of nutrition label in Experiment 2.

Measurements	Label	Without Time Constraints			With Time Constraints		
		Mean	SD	95% CI	Mean	SD	95% CI
Response time	NuVal	3410.47	290.70	[2823.81, 3997.13]	1207.48	40.67	[1125.27, 1289.68]
	5-CNL	3647.99	255.36	[3132.65, 4163.32]	1413.85	38.82	[1335.39, 1492.31]
	TL	2914.42	214.43	[2481.67, 3347.16]	1265.16	34.18	[1196.09, 1334.24]
	DV	4059.87	286.91	[3480.87, 4638.87]	1414.81	62.64	[1288.21, 1541.42]
Number of times choosing labels	NuVal	11.84	1.96	[7.81, 15.88]	14.80	1.72	[11.24, 18.36]
	5-CNL	13.64	1.98	[9.56, 17.72]	13.56	1.40	[10.67, 16.45]
	TL	11.84	1.73	[8.27, 15.41]	21.08	1.39	[18.22, 23.95]
	DV	12.56	1.76	[8.92, 16.20]	8.84	1.13	[6.50, 11.18]

## Data Availability

The datasets generated during and/or analyzed during the current study are available from the corresponding author on reasonable request. The data are not publicly available due to privacy.
